# Use of medicinal plants by breast cancer patients in Algeria

**DOI:** 10.17179/excli2015-571

**Published:** 2015-11-20

**Authors:** Bachir Benarba

**Affiliations:** 1 Laboratory Research on Biological Systems and Geomatics, Faculty of Nature and Life, University of Mascara, Algeria

## Dear Editor,

Cancer is a leading cause of death worldwide accounting for 15 % of all deaths. Breast cancer has been found to be the most common malignancy in females accounting for 36.9 % of the total female cancers in West Algeria (Benarba et al., 2014[1]). In recent years, a high prevalence of the use of alternative medicine and especially medicinal plants among cancer patients has been documented (Tazi et al., 2013[3]). No study has been published previously regarding the use of medicinal plants by Algerian cancer patients. The present study aimed to assess and document the use of medicinal plants among breast cancer patients living in North West Algeria.

Of one hundred fifty patients invited to participate in the present study, sixty nine gave available data about use of medicinal plants. All of them were women, and their mean age was 47 years (age: 47.49 ± 11.07 years). Interestingly, forty-three patients (62 %) were aged 20 - 49 years. Most (81 %) of the population study were married. Regarding intellectual level, twenty nine patients (42 %) were illiterate, twenty four (35 %) followed secondary or higher education, and fourteen (20 %) had primary education level. Of the interviewed patients, fifty five (72.7 %) were housewives and stated that they exercised no professional activity even at home (being housewife in Algeria does not mean being without occupation), and fourteen (20.2 %) were professionals (administration, education and nursing). 

Regarding use of medicinal plants, 43.4 % of breast cancer patients enrolled in the present study used medicinal plants alone or mixed with other plants and/or non-plants-products. Fourteen of them reported having had a beneficial effect of the use of plants during their illness, against twelve who have felt no effect. In addition, three of them have experienced side effects including diarrhea, nausea and in one case, kidney problems. Two patients used vegetal formulation of unknown plants, bought from Oman (Middle East country). These formulations are purchased at prices ranging from 200 - 500 euro through an Arabic TV channel promoting alternative medicine services and products. Two other patients used similar local formulations. The reported plants are given in Table 1(Tab. 1). 

We found that* Aristolochia longa *L. was the most frequently used by breast cancer patients (31.9 %), followed by *Berberis vulgaris L. *(27.6 %) and *Atriplex halimus *(14.9 %). It is the first time that *Berberis vulgaris* L. and *Atriplex halimus* are reported to be used to treat cancer in North Africa. Most of cited species (except *A. longa *and *A. halimus*) are used as mixtures. Honey remains the preferred adjuvant added to different plant species. Addition of honey aims to improve the acceptability of certain plants having a bitter taste unbearable such as *Aristolochia longa *L. 

We must emphasize that *Aristolochia longa* is a toxic plant, due to the presence of aristolochic acids. In a recent study, we have reported that in postmenopausal women newly diagnosed for breast cancer, the intake of *A. longa *roots resulted in renal function problems and high bone resorption, maybe due to the reduction in renal function caused by the aristolochic acids contained in the roots (Benarba et al., 2012[2]). We have demonstrated the anticancer activity of an aqueous extract of *A. longa* roots against breast cancer HBL-100 and MDA-MB-231 cell lines (data not published). Interestingly, the extract (free of aristolochic acids) was safe when administered orally in Wistar rats in a single dose. 

Our study highlights the fact that about half of Algerian breast cancer patients use medicinal plants for “treating” their illness, and that studying the effectiveness and the side effects of such “traditions” should be carried out, urgently. The integration of medicinal plants with conventional therapy of breast cancer could be of great importance in terms of healing. Nevertheless, extensive studies on their effectiveness are essential before use.

## Conflict of interest

The author declares that he has no conflict of interest.

## Figures and Tables

**Table 1:**
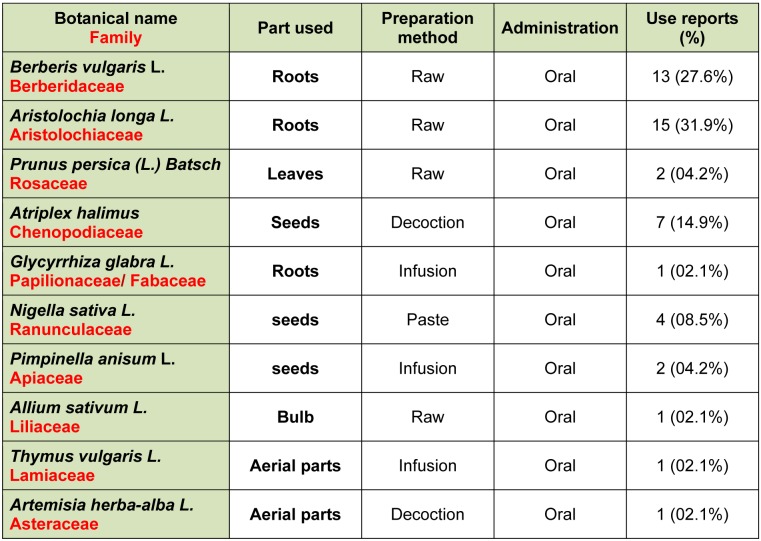
Medicinal plants used by breast cancer patients
